# The Mobile Phone Affinity Scale: Enhancement and Refinement

**DOI:** 10.2196/mhealth.6705

**Published:** 2016-12-15

**Authors:** Beth C Bock, Ryan Lantini, Herpreet Thind, Kristen Walaska, Rochelle K Rosen, Joseph L Fava, Nancy P Barnett, Lori AJ Scott-Sheldon

**Affiliations:** ^1^ Centers for Behavioral and Preventive Medicine The Miriam Hospital Providence, RI United States; ^2^ Department of Psychiatry and Human Behavior Alpert School of Medicine Brown University Providence, RI United States; ^3^ Department of Behavioral and Social Sciences School of Public Health, Brown University Providence, RI United States; ^4^ Department of Public Health University of Massachusetts Lowell Lowell, MA United States; ^5^ Department of Behavioral and Social Sciences Center for Alcohol and Addiction Studies School of Public Health, Brown University Providence, RI United States

**Keywords:** mobile phone, psychometrics, assessment, measure

## Abstract

**Background:**

Existing instruments that assess individuals’ relationships with mobile phones tend to focus on negative constructs such as addiction or dependence, and appear to assume that high mobile phone use reflects pathology. Mobile phones can be beneficial for health behavior change, disease management, work productivity, and social connections, so there is a need for an instrument that provides a more balanced assessment of the various aspects of individuals’ relationships with mobile phones.

**Objective:**

The purpose of this research was to develop, revise, and validate the Mobile Phone Affinity Scale, a multi-scale instrument designed to assess key factors associated with mobile phone use.

**Methods:**

Participants (N=1058, mean age 33) were recruited from Amazon Mechanical Turk between March and April of 2016 to complete a survey that assessed participants’ mobile phone attitudes and use, anxious and depressive symptoms, and resilience.

**Results:**

Confirmatory factor analysis supported a 6-factor model. The final measure consisted of 24 items, with 4 items on each of 6 factors: Connectedness, Productivity, Empowerment, Anxious Attachment, Addiction, and Continuous Use. The subscales demonstrated strong internal consistency (Cronbach alpha range=0.76-0.88, mean 0.83), and high item factor loadings (range=0.57-0.87, mean 0.75). Tests for validity further demonstrated support for the individual subscales.

**Conclusions:**

Mobile phone affinity may have an important impact in the development and effectiveness of mobile health interventions, and continued research is needed to assess its predictive ability in health behavior change interventions delivered via mobile phones.

## Introduction

Mobile phones have shown promise as an effective delivery tool for health behavior change and disease management [1-6], and may be particularly well suited for interventions designed for young adults and adolescents. This population uses texting, apps, and other phone-based applications regularly, a trend that is particularly strong among younger age groups [7]. When developing an intervention that is delivered through mobile devices, it is important to consider how an individual uses his/her mobile phone, as mobile phone use may influence the receptivity to, and ultimately the efficacy of, mobile health (mHealth) programs and interventions [8,9].

There are several published measures that assess the use of technology, including excessive mobile phone use and Internet addiction [10-13]. These measures are largely derived from the addictions and psychopathology literature, and are intended to measure problematic use of technology within a conceptual framework of use-as-pathology [13-15]. Problematic psychological constructs that have been linked to mobile phone use include impulsivity [11], depression [14], and anxiety [14]. However, mobile phones can serve many positive functions. For example, many apps now exist to help people track health behaviors (eg, exercise, weight loss) and manage medical conditions, including diabetes and asthma [16-18]. Mobile phones also serve positive functions, including increasing efficiency and productivity at work, and improving connectivity to social networks, family, and friends [19,20]. However, to our knowledge no measures assess mobile phone and technology use that include items reflective of these positive elements. Therefore, the goals of this study were to expand, revise, and validate the psychometric properties of the Mobile Phone Affinity Scale (MPAS) based on a version that we previously developed in a study of community college students [21]. The current study employs a national cross-sectional survey of adults living in the United States to identify important constructs related to mobile phone use, develop scales to measure these constructs, evaluate the internal consistency of the constructs, and establish the validity of the newly revised instrument.

## Methods

### Questionnaire Development

In the initial development of the MPAS, factors that may be associated with mobile phone use (and more broadly technology use) were identified by conducting a search of the relevant literature using PubMed. Identified constructs included social connectivity, dependence, addiction, mood, and continuous use [21]. To better assess positive functions of mobile phone use, the same search procedures were used in this study to expand the MPAS to include constructs related to empowerment, safety, and usefulness in the domains of social and personal use, and work and school-related use. Individuals from our research team independently wrote a series of items for each of these three additional constructs in English at a sixth grade reading level. The entire research team reviewed the items to determine face validity. Any confusing or ambiguous items were edited and duplicates were deleted. Instructions and response format were also reviewed for clarity. The resulting instrument contained 57 items measuring 7 constructs, with 6-to-9 items per construct, and used a Likert-type scale response format. This initial draft of the revised MPAS was then pretested with eight adults to confirm item clarity and comprehension before administering the measure to a larger sample.

### Psychometric Testing

To test the MPAS, we conducted a national cross-sectional survey between March 25 and April 1, 2016, to assess respondents’ mobile phone use, attitudes toward their mobile phone, current mood, demographic information, and other characteristics. Participants were registered users (*workers*) of Amazon Mechanical Turk (MTurk). We used MTurk to recruit for this survey since research has shown it to be a fast, inexpensive, reliable, and useful approach to collect data from a large and ethnically diverse sample [22-26].

To participate in the study, workers were redirected through the MTurk website to our project survey website, which presented detailed information about the study and an informed consent document. Interested workers who indicated their consent were then linked to the screening questions to assess eligibility. Workers were eligible to participate in the study if they were 18 years of age or older, lived in the United States, could read fluently in English, and owned a mobile phone. If eligible, participants were then directed to the full survey, which asked questions about their mobile phone use, attitudes toward their mobile phone, their current mood, and demographic information.

The online survey was managed using a secure Web-based application, Research Electronic Data Capture [27], hosted in our institution’s Information Services department. Participants were compensated US $1 upon completing the survey. No identifying information was asked of participants, thus keeping their responses anonymous. The informed consent process, assessment of eligibility, and completion of the surveys took an average of 17 minutes to complete. This study was approved by the Institutional Review Board at The Miriam Hospital.

### Measures

#### Mobile Phone Affinity Scale

The initial MPAS consisted of 57 statements about mobile phone use. Participants were asked to report how true each statement was for them, using a 5-point Likert-type response format (1=not at all true to 5=extremely true).

#### Other Measures

In addition to the MPAS, participants responded to demographic questions regarding age, gender, race, ethnicity, education, employment status, and marital status. Participants also responded to questions about their mobile phone (ie, *is it a smartphone?*), the types of activities and apps used on the mobile phone, and the frequency with which they used text messaging.

Previous research has reported increased levels of anxiety, depression, and impulsivity associated with problematic mobile phone use [11,14], and since the MPAS was designed to assess both negative and positive constructs associated with mobile phone use, we included measures of these constructs to validate MPAS subscales. These measures were administered as part of the online questionnaire and included: (1) *symptoms of anxiety*, which were assessed using the 20-item State-Trait Anxiety Inventory (STAI) [28] which is scored on a 4-point scale (1=almost never to 4=almost always), with higher scores indicating higher levels of anxiety; (2) *depressive symptoms*, which were measured using the 10-item Centers for Epidemiological Studies Depression Scale (CESD-10) [29] scored on a 4-point scale (0=rarely/none of the time to 4=most/all of the time); and (3) *impulsiveness*, which was assessed using the 30-item Barratt Impulsiveness Scale (BIS-11) [30] scored on a 4-point scale (1=rarely/never to 4=almost always/always), with higher scores indicating greater impulsiveness. In addition, to differentiate subscales within the MPAS that we hoped would assess more positive and/or beneficial applications of mobile phone use, we chose *psychological resilience* as a positive psychological construct. We assessed this parameter using the 6-item Brief Resilience Scale (BRS) [31] scored on 5-point scale (1=strongly disagree to 5=strongly agree), with higher scores indicating greater psychological resilience.

### Statistical Analyses

First, we used summary statistics (means, standard deviations, and frequencies) to describe the sample characteristics and measures for the entire sample. Second, a preliminary analysis was conducted to examine the MPAS item characteristics, and psychometric analyses were then conducted using confirmatory factor analysis (CFA) in Mplus Version 7.3 [32]. Model fit was evaluated based on the minimum fit function chi-square statistic, the comparative fit index (CFI; [33]), the nonnormed or Tucker-Lewis index (TLI; [34]), the root means square error of approximation (RMSEA; [35]), the standardized root mean square residual (SRMR; [36]), and the model chi-square. Respecifications to the model were guided by theory, in combination with modification indices that are part of the statistical output, and designed to produce a brief measure of mobile phone attachment that is suitable for use in field research. Third, Cronbach alpha [37], a measure of internal consistency reliability, was estimated for each subscale. Finally, to test for concurrent validity of the final measure, we examined the association between MPAS subscales and measures of anxiety, depression, and impulsivity.

## Results

### Participants

Of the 1241 MTurk workers who were redirected to our survey website, 1.05% (13/1241) never completed the informed consent process, 5.32% (66/1241) of the potential participants completed the informed consent process but were deemed to be ineligible for the study, and 8.30% (103/1241) of the participants did not complete the survey. The data for one additional participant was corrupted and removed from the analyses.

Our analyses were restricted to the 1058 participants who agreed to participate in the study and completed all aspects of the survey. Participants were predominately white (877/1058; 82.89%). Men accounted for 49.91% (528/1058) of the participants, and women comprised 50.09% (530/1058) of the sample. Participants were between 18 and 87 years of age (mean 32.5, standard deviation 10.3). Table 1 provides the complete demographic information for the sample.

**Table 1:** Demographics for the overall sample (N=1058).

Sample Characteristic		Proportion, % (n)
**Sex**	
	Male	49.91 (528)
	Female	50.09 (530)
**Race**	
	White	82.89 (877)
	Black	6.14 (65)
	Asian	5.01 (53)
	Native Hawaiian	0.47 (5)
	Pacific Islander	0.28 (3)
	Other	1.51 (16)
	Multiple	3.69 (39)
**Ethnicity**	
	Hispanic	10.02 (106)
	Non-Hispanic	89.98 (952)
**Marital status**	
	Single	51.13 (541)
	Married	39.89 (422)
	Separated	1.61 (17)
	Divorced	6.62 (70)
	Widowed	0.76 (8)
**Census region**	
	Northeast	18.53 (196)
	South	39.13 (414)
	Midwest	21.17 (224)
	West	20.70 (219)
	Pacific	0.47 (5)
**Education**	
	High school or less	10.87 (115)
	Some college	40.36 (427)
	College degree or above	48.68 (515)
**Student**	
	Yes, full-time	14.37 (152)
	Yes, part-time	6.24 (66)
	No	79.30 (839)
**Employed**	
	Yes, full-time	57.09 (604)
	Yes, part-time	20.42 (216)
	No	22.40 (237)
**Income**	
	<$25,000	17.77 (188)
	$25,000-$49,999	32.42 (343)
	$50,000-$99,999	34.50 (365)
	>$100,000	11.53 (122)
	Missing	3.78 (40)
**Living arrangement**	
	Alone	18.34 (194)
	Spouse/partner	30.91 (327)
	Adult roommate	9.36 (99)
	Parents	10.40 (110)
	Child(ren)	3.78 (40)
	Spouse/partner and child(ren)	22.59 (239)
	Multiple	4.63 (49)

### Dimensional and Internal Validity Analyses

Preliminary item level analyses were conducted to examine individual item means, standard deviations, skew, and kurtosis in each of the 57 items, and the results were judged adequate to proceed with the dimensional analysis. An initial CFA model was fit using the full 57-item variable set and specified 7 correlated factors, with each of the items only allowed to load and be freely estimated on its hypothesized factor. This initial model fit poorly (χ^2^_1,518_=9375.0, RMSEA=.07, CFA=.79, TLI=.78, SRMR=.071). The model fit was improved by removing items with poor loadings (<.4), very high cross-factor error correlations, or potentially high cross-factor loading, which indicated a complex item. One further adjustment included collapsing the initially posited separate factors of safety and empowerment into a single factor, based on the modification indices and conceptual similarity of the item content.

The final model (χ^2^_237_=1100.9, RMSEA=.059, CFA=.941, TLI=.931, SRMR=.042) represented a parsimonious and balanced solution with 6 correlated factors, each measured by 4 items, creating a final measure consisting of 24 items (Multimedia Appendix 1). This 24-variable solution fit very well based on alternative criteria suggested by Hu and Bentler [38] and did not use any superfluous adjustments, such as freeing error covariance parameters or allowing variables to load on additional factors, to achieve the final improved model fit. Individual item loadings were high for all items on their respective factors (range=0.57-0.87, mean 0.75; see Table 2), and internal consistency reliability was also very good for each factor, as measured by Cronbach alpha (range=0.76-0.88, mean 0.83; see Table 2). The disattenuated correlations for the latent constructs are presented in Table 3.

Concurrent validity analyses examined correlations between each MPAS subscale and measures of depressive symptoms (CESD-10), anxiety (STAI), resilience (BRS), and impulsiveness (BIS). Depressive symptoms, anxiety, and impulsivity were significantly correlated with *Anxious Attachment*, *Addiction*, and *Continuous Use* subscales, but not with the *Connectedness*, *Productivity*, or *Empowerment* subscales. Resilience was significantly negatively correlated with scores on the *Addiction* and *Anxious Attachment* subscales and positively correlated with the *Productivity* subscale (Table 4).

**Table 2:** Confirmatory factor analyses, item loadings, and Cronbach coefficient alpha for 6 correlated factors that were modeled using the whole sample.

Parameter	Connectedness	Productivity	Empowerment	Anxious Attachment	Addiction	Continuous Use
My phone helps me keep track of my social life	.76					
When it comes to my health or social life, my phone is my personal assistant	.71					
My phone helps me stay close to my friends and family	.70					
My phone makes it easy to cancel plans with others	.60					
My phone helps me to be more organized at work/school		.74				
I use my phone to connect with my co-workers or other students		.75				
My phone is necessary for work/school		.72				
My phone helps me stay up-to-date with work/school activities		.87				
Having my phone with me makes it easier to leave a risky situation			.73			
I feel in control when I have my phone with me			.81			
My phone gives me a sense of security			.84			
I feel safe when I have my phone with me			.83			
I feel anxious if I don’t have my phone with me				.73		
I feel isolated without my phone				.79		
I feel dependent on my phone				.80		
Without my mobile phone, I feel out of touch with the world				.82		
I find myself occupied on my phone even when I’m with other people					.75	
I find myself occupied with my phone when I should be doing other things					.84	
I find myself engaged with my mobile phone for longer periods of time than I intended					.85	
I would get more work done if I spent less time on my phone					.68	
I read/send text messages from my mobile phone, when I am at work or in class, that are not related to what I am doing						.57
I use my phone all day						.74
I am never bored if I have my phone with me						.61
I rely on my phone 24/7						.79
Cronbach alpha	.78	.85	.88	.86	.86	.76

**Table 3:** Disattenuated correlations among 6 latent constructs from confirmatory factor analyses (upper-right triangle), with the raw summated scale score correlations (lower-left triangle).

	Connectedness	Productivity	Empowerment	Anxious Attachment	Addiction	Continuous Use
Connectedness	–	.74	.82	.77	.66	.81
Productivity	.60	–	.55	.53	.46	.65
Empowerment	.69	.48	–	.78	.47	.69
Anxious Attachment	.63	.45	.68	–	.72	.84
Addiction	.54	.40	.41	.61	–	.78
Continuous Use	.65	.55	.57	.68	.67	–

**Table 4:** Correlations for complete sample among the 6 Mobile Phone Affinity Scale (MPAS) scores and Centers for Epidemiologic Studies Depression Scale (CESD), State-Trait Anxiety Inventory (STAI), Barratt Impulsiveness Scale (BIS-11), and Brief Resilience Scale (BRS).

	Depression (CESD-10)		Anxiety (STAI)		Impulsivity (BIS-11)		Resilience (BRS)	
	r^a^	*P* value	r^a^	*P* value	r^a^	*P* value	r^a^	*P* value
Connectedness	.03	.36	-.00006	.99	.01	.81	.06	.07
Productivity	.02	.56	-.04	.20	-.04	.21	.09	<.001
Empowerment	.02	.51	.03	.39	-.01	.64	.00	.95
Anxious Attachment	.13	<.001	.15	<.001	.13	<.001	-.12	<.001
Addiction	.25	<.001	.18	<.001	.29	<.001	-.15	<.001
Continuous Use	.10	<.001	.08	.01	.14	<.001	-.03	.28

^a^r=Pearson product-moment correlation coefficient

## Discussion

This study developed, revised, and validated the MPAS, a multi-dimensional survey instrument with strong internal consistency and high item factor loadings across 6 distinct subscales. The MPAS provides a measure of an individual’s relationship to his/her mobile phone with positive (*Connectedness*, *Productivity*, *Empowerment*), negative (*Anxious Attachment*, *Addiction*), and neutral (*Continuous Use*) valences. Results showed that the subscales we conceived of as positive (eg, *Connectedness*) were not correlated with depressive symptoms, anxiety, or impulsivity, while both negative subscales (*Anxious Attachment*, *Addiction*) were correlated with these features. Personal resilience, a positive characteristic, was significantly and positively correlated with affinity for the mobile phone for *Productivity* purposes and negatively correlated with both negative subscales. This finding differentiates the MPAS from other instruments in that both impulsivity and anxiety have been shown to be associated with mobile phone use in studies of mobile phone addiction or dependence [39-41]. However, the *Connectedness* subscale was not correlated with resilience, which is reasonable given that personal relationships with others can help promote personal resilience [42,43], but can also place a burden on individuals [44]. Thus, no clear positive or negative association with resilience would be expected among those with high affinity for using their mobile phone for social connections.

In this revision of the MPAS, we have succeeded in our goal of creating a scale that represents both positive and negative aspects of increased use of mobile technology. Using the previous 4-subscale version, most scales were correlated with anxiety-related and depressive symptoms [21], while a much clearer differentiation was observed in the present 6-subscale version. In the current revised version, only *Continuous Use*, *Anxious Attachment,* and *Addiction* were associated with depressive symptoms, while use of mobile phones for *Connectedness*, *Productivity,* and *Empowerment* were not. Furthermore, in the current version of the MPAS, the *Continuous Use* subscale is correlated with anxiety, but less strongly than the *Anxious Attachment* or *Addiction* subscales, suggesting that some level of anxiety can be a positive, functional trait, by enhancing attentiveness to important things (eg, making sure to bring your phone with you or ensuring the battery is charged).

### Limitations

Ultimately, we anticipate the value of the MPAS will be to provide predictive value for mHealth interventions, necessitating the assessment of whether scores on the MPAS (or any of its subscales) relate significantly to outcomes of interventions. However, this instrument was not developed in the context of an intervention study, and it is a limitation of this study that we did not assess other factors (eg, intentions to change health behaviors or outcome expectations) that may have served as a proxy for intervention outcomes. A second important limitation relates to the nature of the study sample. Although MTurk workers tend to be racially and ethnically diverse, they are likely to be more technologically savvy than most mobile phone users, and may not be representative of the overall US population in that regard. Thus, it is possible that the mean value of any individual MPAS subscale may differ between the MTurk study sample and other US population samples, but the high internal consistency reliability of each subscale, and the associated high item loadings on each subscale, indicate that the instrument has strong internal validity. We expect that this instrument will prove valuable for its intended purposes when used in other adult samples.

### Future Directions

Additional work is needed to examine whether scores on the MPAS or its subscales are predictive of uptake, maintenance, and successful outcomes among individuals who are interacting with a behavioral health intervention delivered (in whole or in part) through mobile technology. It is our hope that the MPAS developed in this study may prove to be a useful indicator of the quality of the individual’s relationship with their mobile phone, and may comprise an important element in understanding the efficacy of mHealth interventions and programs.

There is tremendous growth in technologies that can provide health care and behavioral health interventions through mobile channels, such as apps and text messaging [16-18]. While the qualities of both the intervention and the delivery technology are important, in our quest to understand the effects that these interventions have on behaviors, it is also important to understand the relationship that the individual has with their mobile phone. mHealth involves not only health behaviors, but also those behaviors and attitudes relevant to interacting with mobile technology, and interacting with other people through mobile devices. Given the immediate and reciprocal nature of both health behavior and the behavior of interacting with a mobile device, thought leaders have suggested that our current behavioral health theories may be inadequate, particularly as mHealth interventions become increasingly interactive and adaptive [45]. Several papers have called for an expansion of our understanding of how interacting with a mobile device impacts health behavior [46,47]. Research is needed that can contribute to new theories regarding the interaction between mobile technology use, mHealth interventions, and behavior change.

## Appendix

Multimedia Appendix 1 Final 24-item Mobile Phone Affinity Scale.

## Abbreviations

BIS-11: Barratt Impulsiveness Scale
BRS: Brief Resilience Scale
CESD-10: Centers for Epidemiologic Studies Depression Scale
CFA: confirmatory factor analysis
CFI: Comparative Fit Index
mHealth: mobile health
MPAS: Mobile Phone Affinity Scale
MTurk: Amazon Mechanical Turk Service
RMSEA: root mean square error of approximation
SRMR: standardized root mean square residual
STAI: State-Trait Anxiety Inventory
TLI: Tucker-Lewis Index
