# Supplementary material for: The Mobile Phone Affinity Scale: Enhancement and Refinement
Source: JMIR Mhealth Uhealth. 2016 Dec 15;4(4):e134. doi: 10.2196/mhealth.6705 (PMC5200845; doi:10.2196/mhealth.6705)
Supplement: Multimedia Appendix 1 [file mhealth_v4i4e134_app1.pdf]

Mobile Phone Affinity Scale (MPAS)

Record ID ______________________________

INSTRUCTIONS: People use their mobile phone for different things, some are more important than others. Please use the 1-5 scale provided ("Not at all true" to "Extremely true") to rate how TRUE for YOU the following statements are.

Don't spend too much time on any statement. Answer quickly and honestly.

	Not at all true	A little true	Somewhat true	Very true	Extermely true
My phone helps me keep track of my social life.	○	○	○	○	○
I use my phone to connect with my co-workers or other students.	○	○	○	○	○
I would get more work done if I spent less time on my phone.	○	○	○	○	○
When it comes to my health or social life, my phone is my personal assistant.	○	○	○	○	○
I find myself occupied with my phone when I should be doing other things.	○	○	○	○	○
I feel safe when I have my phone with me.	○	○	○	○	○
I feel dependent on my phone.	○	○	○	○	○
I feel anxious if I don't have my phone with me.	○	○	○	○	○
I read/send text messages when I am at work or in class that are not related to what I am doing.	○	○	○	○	○
I find myself occupied on my phone even when I'm with other people.	○	○	○	○	○
Having my phone with me makes it easier to leave a risky situation.	○	○	○	○	○
I rely on my phone 24/7.	○	○	○	○	○
My mobile phone helps me to stay up-to-date with work/school activities.	○	○	○	○	○
I feel isolated without my phone.	○	○	○	○	○

I feel in control when I have my phone with me.	○	○	○	○	○
My phone is necessary for work/school.	○	○	○	○	○
Without my mobile phone I feel out of touch with the world.	○	○	○	○	○
I am never bored if I have my phone with me.	○	○	○	○	○
My phone helps me stay close to my friends and family.	○	○	○	○	○
My phone makes it easy to cancel plans with others.	○	○	○	○	○
I use my phone all day.	○	○	○	○	○
My phone gives me a sense of security.	○	○	○	○	○
My phone helps me to be more organized at work/school.	○	○	○	○	○
I find myself engaged with my mobile phone for longer period of time than I intended.	○	○	○	○	○
